# Epigenome-wide DNA methylation and spontaneous preterm birth among pregnant black women

**DOI:** 10.1186/s13148-026-02151-8

**Published:** 2026-05-24

**Authors:** Tingting Zhao, Yihong Zhao, Paolo Reho, Goleen Samari, Ronald Wapner, Arielle Hazi, Haotian Wu, Veronica Barcelona

**Affiliations:** 1 School of Nursing, Columbia University, 560 W 168Th St, New York, NY 10032 USA; 2 Mailman School of Public Health, Columbia University, New York, NY USA; 3 Population and Public Health Sciences, Keck School of Medicine, University of Southern California, Los Angeles, CA USA; 4 Department of Obstetrics and Gynecology at, Columbia University Irving Medical Center, Columbia University, New York, NY USA

**Keywords:** Spontaneous Preterm Birth (sPTB), Epigenome-wide Association Studies (EWAS), Pregnant black women

## Abstract

**Background:**

Preterm birth (PTB, < 37 weeks of gestation) is a major public health concern in the United States, with Black women experiencing a higher incidence compared to White women. Although some studies have identified social, medical, and obstetric risk factors for PTB, the biological mechanisms underlying spontaneous PTB (sPTB) risk remain unclear. We conducted a secondary analysis using data from Black participants enrolled in the Nulliparous Pregnancy Outcomes Study: Monitoring Mothers-to-be (nuMoM2b), (n = 1073) from 2010 to 2013. Peripheral whole blood samples were collected from all participants between 6 + 0/7 and 13 + 6/7 weeks of gestation. Demographic, behavioral and clinical data were gathered through surveys, and pregnancy outcomes were obtained through chart abstraction. We used the Infinium Methylation EPIC v2.0 BeadChip for epigenome-wide association (EWAS) of DNA methylation and sPTB.

**Results:**

We identified 19 differentially methylated CpGs associated with sPTB, 17 CpGs were mapped to 27 annotated genes including *CASR* (cg19108881), *KLF2/ KLF2-DT* (cg18473733), *SNX14/SYNCRIP* (cg25689730), *FBXO2* (cg04872402)*, HPAT5/LOC102725048* (cg12798411), *RTN4RL1/LOC105371486* (cg03098704)*, SETD4* (cg25826287)*, SLC25A16* (cg15165108), *CLYBL/CLYBL-AS3 (*cg12328625*), HES7* (cg09601704)*, SLC67A1/ SLC67A1-AS (*cg05919744*), ATP2A3 (*cg12120292*), PSMB8/PSMB8-AS1/ PSMB9, TAP2* (cg24031377)*, SLCO3A1* (cg05111081), *KIAA1671* (cg26144263), *ALDH4A1* (cg 12,092,708), and *GUCD1* and *SNRPD3* (cg11895717)*.* Functional enrichment analysis revealed molecular functions related to enzymatic protein degradation (threonine peptidases) and energy-dependent transport of substances across membranes and biological processes including cellular transport and vascular regulation, which may influence early embryonic development and contribute to sPTB risk.

**Conclusions:**

Our study identified potential epigenetic alterations associated with sPTB risk and highlighted candidate genes, molecular functions, and biological processes that may serve as predictors in pregnant Black women. Future research should examine larger samples of Black women and include social determinants of health (SDOH) such as individual- and structural- racism to explain racial disparities in methylation patterns. There is a need for larger studies that examine the interactions between SDOH and epigenomic mechanisms underlying adverse birth outcomes.

**Supplementary Information:**

The online version contains supplementary material available at 10.1186/s13148-026-02151-8.

## Introduction

Preterm birth (PTB, < 37 weeks’ gestation) is a major public health concern in the United States, with Black women experiencing a 1.5-fold higher incidence compared to White women [1]. PTB infants born to Black women have an increased risk of poor health outcomes, including short- and long-term developmental impairments [2, 3]. Although racial disparities exist for both spontaneous PTB (sPTB) and medically indicated PTB, sPTB accounts for the majority of PTB cases (> 50%), and its etiology remains largely unknown [4–6]. While some studies have associated maternal socioeconomic factors, such as age, education, income, and behaviors like smoking with PTB risk, findings have been inconsistent [7]. Moreover, these factors do not fully explain the persistent racial disparities in sPTB risk.^8^

Emerging research has begun to investigate biological pathways that may be influenced by social and environmental factors as an explanation for racial inequities [9]. One potential mechanism is DNA methylation (DNAm), an epigenetic modification that regulates gene expression without altering the DNA sequence and is sensitive to maternal environmental and psychosocial stressors [10]. The most commonly studied form of DNAm is 5-methylcytosine, which involves the addition of a methyl group to the fifth carbon of a cytosine residue within a CpG dinucleotide [11]. Previous studies have shown that prenatal stress, socioeconomic disadvantage, and exposure to racism are associated with differential DNAm patterns in genes related to inflammation, stress response (e.g., *NR3C1, NR3C2, BDNF, CRH, CRHBP, FKBP5, HSD11B2, SLC6A4, CRHR1, CRHR2, and IGF1*), and neurodevelopment (e.g., *BDNF*, *OXTR, MAPT, and CLU)*. [12–16] These findings suggest that DNAm may serve as a molecular pathway linking maternal stress exposure to adverse perinatal outcomes.

Despite these advances, few studies have specifically investigated DNAm signatures associated with sPTB among pregnant Black women. To address this gap, we conducted an epigenome-wide association study (EWAS) using peripheral whole blood samples of pregnant Black women to identify DNAm patterns associated with sPTB. We hypothesized that differential methylation in maternal prenatal stressor-related genes would be associated with sPTB risk, offering new insights into the biological mechanisms underlying racial disparities in sPTB.

## Methods

### Study participants

The Nulliparous Pregnancy Outcomes Study: Monitoring Mothers-to-be (nuMoM2b) study was a prospective longitudinal cohort study of nulliparous women between 6 (6 + 0/7) and 13 (13 + 6/7) weeks gestation who were recruited for participation (N = 10,038) from eight academic medical centers between 2010 and 2014 [17]. Verbal or written informed consent was obtained from pregnant women during recruitment, and the study included the collection of behavioral and demographic data, biospecimens and clinical data, and electronic health records data for birth outcomes. Haas [17] We conducted a secondary analysis of all Black participants (n = 1073) with stored DNA from nuMoM2b for the present analysis. The study procedures were approved by the Columbia University Institutional Review Board (#AAAU0215).

Maternal demographic data including age, body mass index (BMI), education, and smoking history during pregnancy were collected from surveys during study visits. Infant gestational age at birth and sex were retrieved from electronic chart review. Inclusion criteria for the parent study included viable singleton gestation pregnant women ≥ 13 years old with a gestational age between 6 weeks 0 days (6 + 0/7) and 13 weeks 6 days (13 + 6/7) at the time of enrollment (first trimester). Women were excluded from participation if they had known fetal malformations, experienced a loss before 20 weeks’ gestation, or if they planned to terminate the pregnancy.

### Blood DNA extraction and analysis

**Fig. 1:**
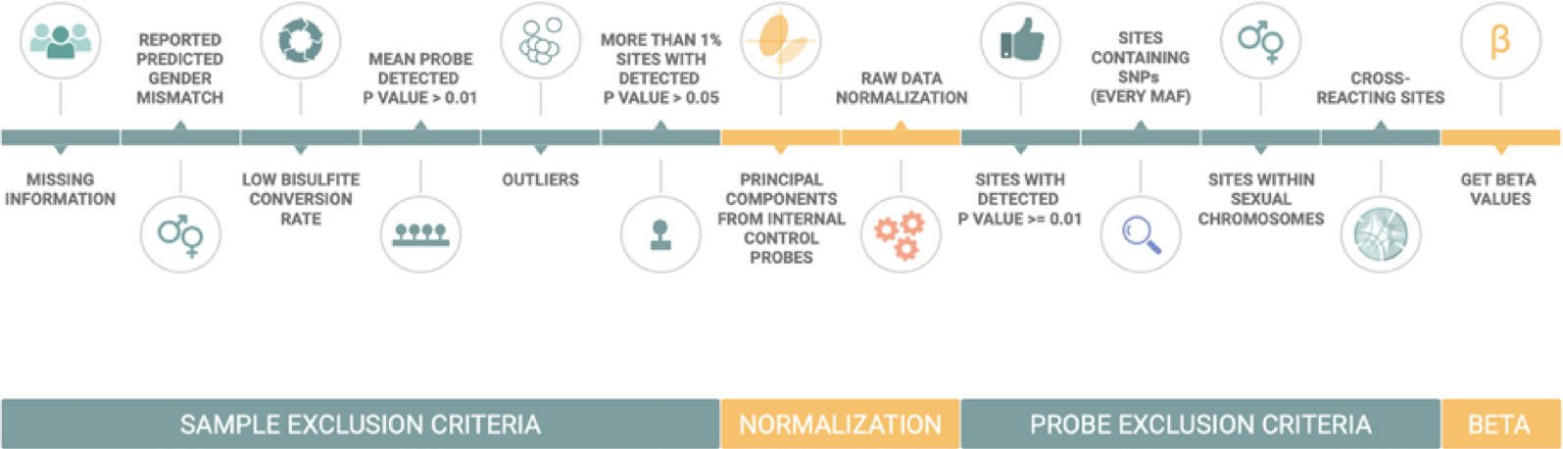
Workflow of the EWAS study. Figure created with BioRender.com

Maternal peripheral whole blood samples (4—8 ml) were collected during the initial study visit, as part of the parent nuMoM2b study (Fig. 1). Haas [17] Genomic DNA was extracted from these samples, stored at − 80 °C, and normalized prior to epigenome-wide DNA methylation analysis. Bisulfite conversion was performed using the EZ-96 DNA Methylation Kit (Zymo Research) following the manufacturer’s instructions. To be included in analyses, samples were required to have a minimum of 350 ng gDNA and to have passed quality control checks. Methylation was measured on the Illumina Infinium MethylationEPIC v2.0 BeadChips at the University of Minnesota Genome Center.

### DNA methylation data quality control, and methylation preprocessing

DNA methylation data were preprocessed using the MethylCallR R package, which offers a standardized, EPICv2-aware pipeline for quality control and probe filtering [18]. Raw IDAT files from 1,073 samples were imported and underwent quality filtering based on established criteria (Fig. 1). At the sample level, low-quality samples were excluded if the median log₂ signal intensity fell below 10.5, consistent with thresholds used in the minfi package. Samples were also removed if more than 1% of probes failed detection (i.e., detection *P*-value > 0.01). At the probe level, probes were removed if they met any of the following criteria:1) detection *P*-value > 0.01 in > 1% of samples; 2) bead count < 3 in > 5% of samples; 3) mapped to non-CG sites; 4) located on chromosome Y; 5)associated with SNPs common in the AFR population (per SeSaMe’s AFR manifest); 6) identified as cross-hybridizing via BLAT; 7) flagged as low-reproducibility in the EPICv2 manifest; or 8) failed to map to GRCh38/hg38 or were mismatched with the SeSaMe manifest. Further, EPICv2-specific duplicated probes were also removed using MethylCallR's internal reference data to ensure cross-platform compatibility. After filtering, 102,737 poor-quality probes and 3 low-quality samples were excluded, yielding 1070 samples and 834,231 high-confidence CpG probes for downstream analyses. CpG site locations are reported based on the GRCh38 (hg38) reference genome.

Normalization was performed using the Noob (normal-exponential out-of-band) method with an offset of 100, excluding XY probe interpolation. Outlier detection via Mahalanobis distance identified 11 samples with aberrant methylation profiles, which were subsequently removed. To adjust for technical variability, known batch variables (e.g., Sentrix barcode, sample section) were corrected using the MeCall.RemoveBatch() function. Importantly, preterm birth status, our primary phenotype, was preserved during batch correction. Cell-type composition was estimated using MeCall.CellComp(), providing proportions for CD8 + T cells, CD4 + T cells, natural killer (NK) cells, B cells, monocytes, and neutrophils.

### EWAS analysis in PTB

EWAS analyses were conducted using the MeCall.DMP() function, which implements a linear modeling framework via the limma package. Batch-adjusted beta values derived from EPIC v2 arrays served as input for the models. The primary exposure was preterm birth status, which was dichotomized at 37 weeks gestation, and included only spontaneous preterm births. Models were adjusted for relevant covariates, including maternal age, smoking history during pregnancy (any vs. none), educational attainment, BMI at the first prenatal visit, and infant sex assigned at birth. Additionally, we performed sex-specific EWAS analysis. To account for potential technical artifacts and unmeasured confounding, the first 10 principal components derived from the methylation data were included as covariates. Additionally, estimated proportions of major blood cell types were incorporated into the models. Participants with missing covariate data, incomplete methylation profiles, or poor-quality methylation measurements were excluded from the analysis. Differentially methylated positions (DMPs) were identified using empirical Bayes moderated t-statistics. Multiple testing correction was performed using the Benjamini–Hochberg method to control the false discovery rate (FDR).

### Functional annotation

Significant CpGs (FDR < 0.1) annotation was retrieved using the annotatr() package [19]. Functional enrichment analysis was performed using the g:Profiler toolkit (version 0.2.3), considering pathways from multiple sources, including Gene Ontology **(GO)**, KEGG, Reactome, WikiPathways, TRANSFAC, miRTarBase, and the Human Phenotype Ontology [20]. Only pathways with a Bonferroni adjusted *p*-value < 0.05 and involving more than one gene were retained for the analysis.

### Results

In total, 933 pregnant women were included in the study, of whom 6% delivered sPTB (Table 1). Pregnant women who delivered preterm due to sPTB were slightly older compared to those with term births although the findings were not significant. There were no significant differences between the two groups in terms of smoking during pregnancy, maternal education, or BMI categories (i.e., underweight, normal, overweight, obesity I, and obesity II-III). The sex distribution of infants was balanced across both term and sPTB groups, with 50.7% male and 49.3% female.

**Table 1:** Demographic characteristics of pregnant black women by pregnancy outcome

	Full Term (N = 878)	Spontaneous PTB (N = 55)	Total (N = 933)	*p* value
Maternal age				0.255^1^
Mean (SD)	23.37 (5.22)	24.95 (6.70)	23.46 (5.33)	
Range	13.00–42.00	17.00–45.00	13.00–45.00	
Smoked during pregnancy				0.599^2^
No	786 (89.5%)	48 (87.3%)	834 (89.4%)	
Yes	92 (10.5%)	7 (12.7%)	99 (10.6%)	
Education				0.160^2^
High school or less	368 (41.9%)	30 (54.5%)	398 (42.7%)	
Some college or college degree	449 (51.1%)	21 (38.2%)	470 (50.4%)	
Education beyond college	61 (6.9%)	4 (7.3%)	65 (7.0%)	
BMI category				0.140^2^
Underweight	15 (1.7%)	2 (3.6%)	17 (1.8%)	
Normal	316 (36.0%)	24 (43.6%)	340 (36.4%)	
Overweight	225 (25.6%)	6 (10.9%)	231 (24.8%)	
Obesity I	152 (17.3%)	10 (18.2%)	162 (17.4%)	
Obesity II/III	170 (19.4%)	13 (23.6%)	183 (19.6%)	
Infant sex				0.601^2^
Female	431 (49.1%)	29 (52.7%)	460 (49.3%)	
Male	447 (50.9%)	26 (47.3%)	473 (50.7%)	

*BMI* body mass index, *PTB* preterm birth, *SD* standard deviation

^1^Wilcoxon rank sum test

^2^Chi-squared test

### Epigenome identifies differentially methylated probes

**Fig. 2:**
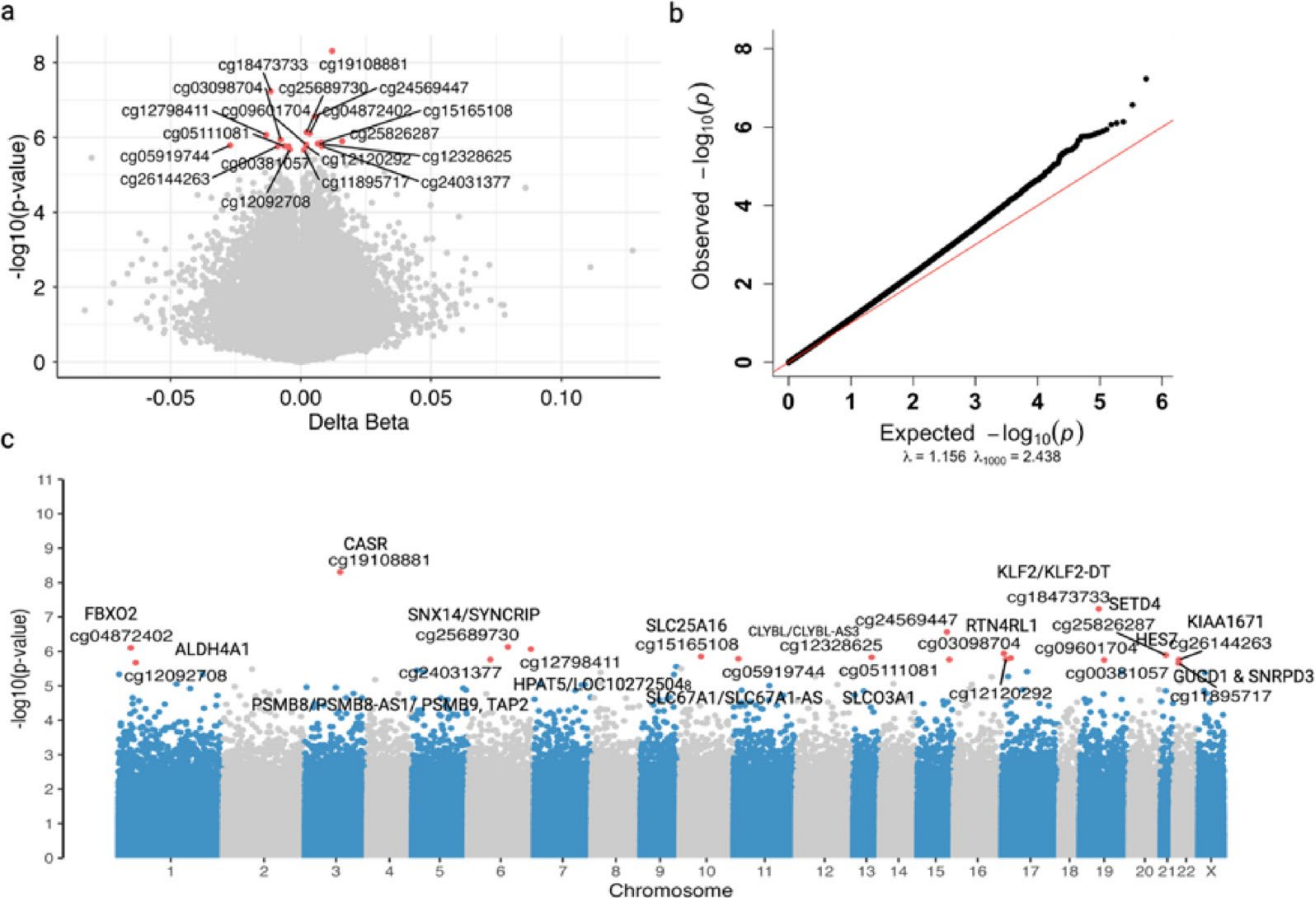
Volcano plot, QQ plot, and Manhattan plot of EWAS results. a. Volcano plot displaying the statistical significance and magnitude of change for all CpG sites included in the spontaneous preterm birth (sPTB) EWAS analysis. Red dots indicate significantly hypermethylated DMPs in sPTB infants compared to term infants. **b.** QQ plot showing the distribution of observed *p*-values and inflation (lambda value). **c.** Manhattan plot illustrating the p-values of tested probes across the genome. Probes surpassing the FDR threshold are shown in red

We identified 19 differentially methylated CpGs (DMCs) suggestively associated with sPTB at an FDR < 0.10 level (Table 2). Of these, two DMPs (i.e., cg19108881 and cg18473733) surpassed the genome-wide significance threshold (FDR adjusted *p* < 0.05) (Table 2) while adjusting for maternal age, BMI, education, and smoking history during pregnancy. Among 19 CpGs, 17 DMPs were mapped to 27 annotated gene regions (Fig. 2a and c), however, these annotations may be located up to 5 kb from the gene and therefore do not necessarily overlap the gene body. These annotated genes are calcium-sensing receptor (*CASR)* (cg19108881, FDR adjusted *p* = 0.004, Δβ = 0.012), Krüppel-like factor 2 (*KLF2) and* KLF2 divergent transcript (*KLF2-DT*) (cg18473733, FDR adjusted *p* = 0.025, Δβ = − 0.012), Sorting nexin 14 (*SNX14*) and Synaptotagmin Binding Cytoplasmic RNA Interacting Protein (*SYNCRIP)* (cg25689730, FDR adjusted *p* = 0.087, Δβ = 0.002), F-Box Protein 2 (*FBXO2*) (cg04872402, FDR adjusted *p* = 0.087, Δβ = 0.003), Human Pluripotency-Associated Transcript 5 (*HPAT5*) and Uncharacterized long non-coding RNA (*LOC102725048*) (cg12798411, FDR adjusted *p* = 0.087, Δβ = − 0.013), Reticulon 4 Receptor Like 1 (*RTN4RL1*) and Uncharacterized long non-coding RNA (*LOC105371486*) (cg03098704, FDR adjusted *p* = 0.087, Δβ = − 0.008), SET Domain Containing 4 (*SETD4*) (cg25826287, FDR adjusted *p* = 0.087, Δβ = 0.016), Solute carrie family 25 member 16 (*SLC25A16*) (cg15165108, FDR adjusted *p* = 0.087, Δβ = 0.008), Citramalyl-CoA Lyase Beta Like (*CLYBL*) and CLYBL Antisense RNA 3 (*CLYBL-AS3*) (cg12328625, FDR adjusted *p* = 0.087, Δβ = 0.007), Hes family BHLH transcription factor 7 (*HES7*) (cg09601704, FDR adjusted *p* = 0.087, Δβ = 0.002), Solute Carrier Family 67 Member 1 (*SLC67A1*) and SLC67A1 Antisense RNA (*SLC67A1-AS*) (cg05919744, FDR adjusted *p* = 0.087, Δβ = − 0.027), ATPase Sarcoplasmic/Endoplasmic Reticulum Ca^2^⁺ Transporting 3 (*ATP2A3*) (cg12120292, FDR adjusted *p* = 0.087, Δβ = 0.002), Proteasome subunit beta type-8 (*PSMB8*), PSMB8 Antisense RNA 1 (*PSMB8-AS1*), Proteasome Subunit Beta 9 (*PSMB9*), and Transporter 2, ATP Binding Cassette Subfamily B Member (*TAP2*) (cg24031377, FDR adjusted *p* = 0.087, Δβ = 0.008), Solute Carrier Organic Anion Transporter Family Member 3A1 (*SLCO3A1*) (cg05111081, FDR adjusted *p* = 0.087, Δβ = − 0.006), KIAA1671 Protein (*KIAA1671*) (cg26144263, FDR adjusted *p* = 0.087, Δβ = − 0.009), Aldehyde Dehydrogenase 4 Family Member A1 (*ALDH4A1*) (cg 12,092,708, FDR adjusted *p* = 0.096, Δβ = − 0.004), and Guanylyl cyclase domain containing 1 (*GUCD1*) and Small Nuclear Ribonucleoprotein D3 Polypeptide (*SNRPD3*) (cg11895717, FDR adjusted *p* = 0.096, Δβ = 0.001). *SLC67A1, SLC67A1-AS, HPAT5, LOC102725048, KLF2, KLF2-DT, KIAA1671, RTN4RL1, LOC105371486, SLCO3A1*, and *ALDH4A1* were hypomethylated, while other 16 genes were hypermethylated in women who delivered preterm infants compared with those who delivered at term (Table 2). Our data demonstrated modest inflation (Lambda = 1.16) (Fig. 2b).

**Table 2:** Differentially methylated probes among pregnant Black women who delivered preterm infants compared with term infants

Probe ID	Chromosome	Position	Gene	Gene region	Δβ	*p* value	Adjusted *p* value
cg19108881	chr3	122,183,663	CASR	TSS1500	0.012	4.93E-09	0.004
cg18473733	chr19	16,326,551	KLF2, KLF2-DT	–	− 0.012	5.87E-08	0.025
cg24569447	chr15	88,769,564	–	–	0.005	2.72E-07	0.076
cg25689730	chr6	85,593,744	SNX14, SYNCRIP	exon_1	0.002	7.35E-07	0.087
cg04872402	chr1	11,645,926	FBXO2	–	0.003	7.85E-07	0.087
cg12798411	chr6	167,235,896	HPAT5, LOC102725048	–	− 0.013	8.61E-07	0.087
cg03098704	chr17	1,957,116	RTN4RL1, LOC105371486	–	− 0.008	1.15E-06	0.087
cg25826287	chr21	36,073,515	SETD4	–	0.016	1.28E-06	0.087
cg15165108	chr10	68,527,740	SLC25A16	TSS1500	0.008	1.40E-06	0.087
cg12328625	chr13	99,865,146	CLYBL, CLYBL-AS3	–	0.007	1.48E-06	0.087
cg09601704	chr17	8,124,109	HES7	TSS200	0.002	1.56E-06	0.087
cg05919744	chr11	2,902,928	SLC67A1, SLC67A1-AS	–	− 0.027	1.64E-06	0.087
cg12120292	chr17	3,963,933	ATP2A3	–	0.002	1.70E-06	0.087
cg24031377	chr6	32,842,249	PSMB8, PSMB8-AS1, PSMB9, TAP2	exon_4	0.008	1.72E-06	0.087
cg05111081	chr15	91,882,266	SLCO3A1	–	− 0.006	1.73E-06	0.087
cg26144263	chr22	24,968,597	KIAA1671	–	− 0.009	1.74E-06	0.087
cg00381057	chr19	29,873,781	–	–	− 0.005	1.77E-06	0.087
cg12092708	chr1	18,902,651	ALDH4A1	TSS200	− 0.004	2.13E-06	0.096
cg11895717	chr22	24,555,095	GUCD1, SNRPD3	exon_1	0.001	2.19E-06	0.096

Chromosome positions are shown relative to the human reference genome (hg38). The *Δβ* values refer to the difference between DNA methylation (*β*-values) in cases compared to controls (e.g., 0.012 indicates that pregnant Black women who delivered preterm infants show a 1.2% increase in DNA methylation compared to term infants). Adjusted *p* value refer to FDR adjusted p values

### DNA methylation profile by infant sex

To explore sex-specific differences in DNA methylation, we performed EWAS including an interaction term between infant sex and birth outcomes (sPTB vs term) among participants with male (sPTB, n = 26; term, n = 447) and female infants (sPTB, n = 29; term, n = 431). Our findings showed only marginally differences between female term and sPTB infants, likely due to the small sample of sPTB participants (Supplementary Table 1). The annotated genes include *KLF2* (cg18473733, FDR adjusted *p* value = 0.089, Δβ = − 0.016), Regulatory Associated Protein of MTOR Complex 1 (*RPTOR*) (cg23063399, FDR adjusted *p* value = 0.089, Δβ = − 0.023), NMDA Receptor Synaptonuclear Signaling and Neuronal Migration Factor (*NSMF*) (cg06077979, FDR adjusted *p* value = 0.089, Δβ = 0.019), and Pre-mRNA Processing Factor 38A (*PRPF38A*) (cg19776520, FDR adjusted *p* = 0.089, Δβ = 0.005). In contrast, one hypermethylated probe, cg00022064 (FDR adjusted *p* value < 0.001, Δβ = 0.045), showed significantly epigenetic differences between male sPTB and term infants (Supplementary Table 2).

Functional enrichment analysis determined pathways associated with sPTB.

**Fig. 3:**
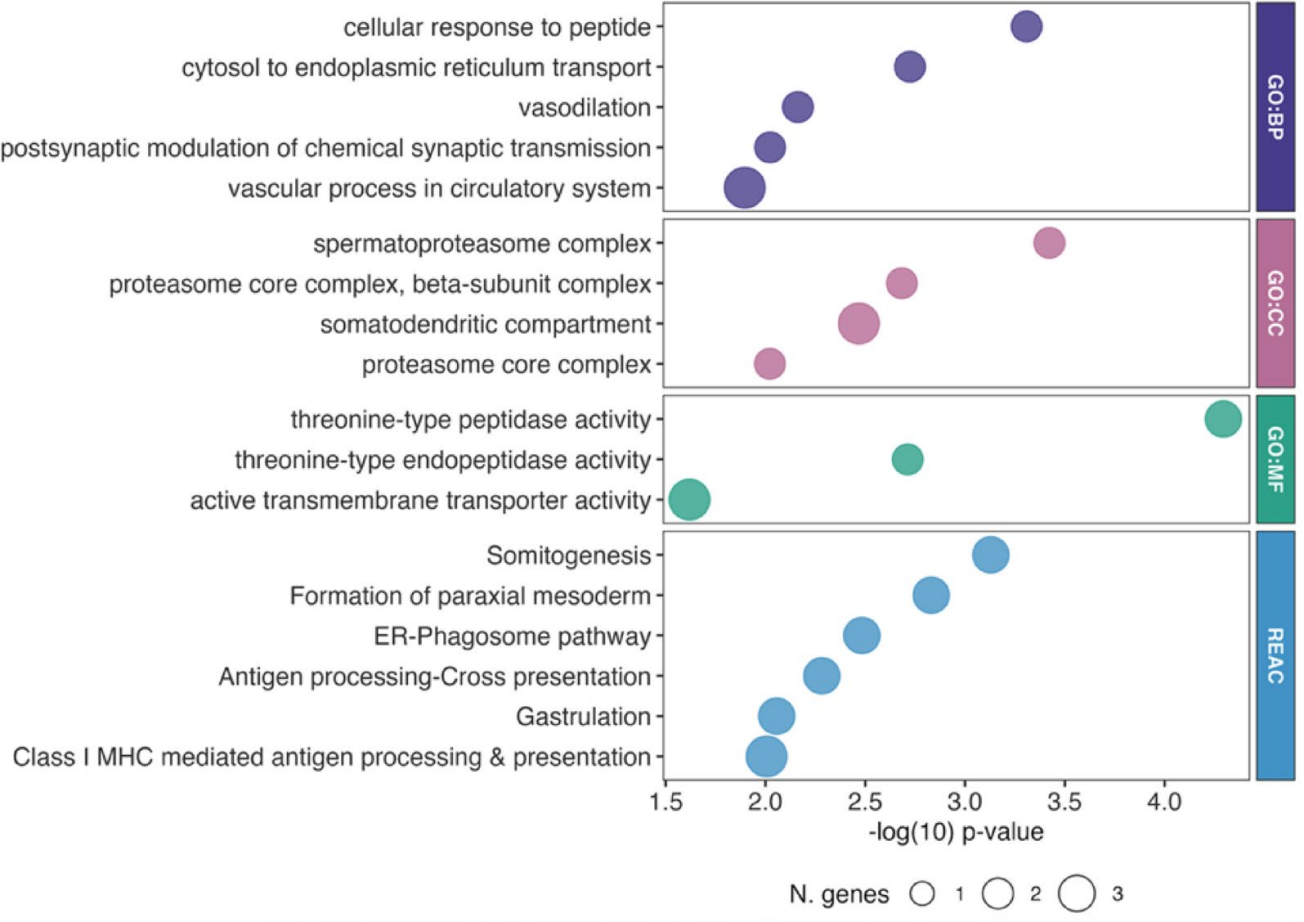
Gene Ontology (GO) enrichment analysis. The x-axis represents statistical significance (− log₁₀ adjusted p-value), and the y-axis lists the GO categories identified in the analysis. Colored dots indicate different functional classifications: purple for biological processes (BP), and pink for molecular functions (MF)

We performed a functional enrichment analysis of differentially methylated genes (Fig. 3). We were able to obtain a list of 27 genes annotated with the 19 DMCs (Table 2). This approach identified five GO biological processes including: cellular response to peptide (GO:1,901,653, FDR adjusted *p* = 4.91E-04), cytosol to endoplasmic reticulum transport (GO:0046967, FDR adjusted *p* = 1.89E-03), vasodilation (GO:0042311, FDR adjusted *p* = 6.88E-03), postsynaptic modulation of chemical synaptic transmission (GO:0099170, FDR adjusted *p* = 9.48E-03), and vascular process in circulatory system (GO:0003018, FDR adjusted *p* = 1.27E-02). The analysis highlighted GO cellular components and molecular functions, including: spermatoproteasome complex (GO:1,990,111, FDR adjusted p = 3.78E-04), proteasome core complex, beta-subunit complex (GO:0019774, FDR adjusted *p* = 2.07E-03), somatodendritic compartment (GO:0036477, FDR adjusted *p* = 3.40E-03), proteasome core complex (GO:0005839, FDR adjusted *p* = 9.50E-03), threonine-type peptidase activity (GO:0070003, FDR adjusted *p* = 5.08E-05), threonine-type endopetidase activity (GO:0004298, FDR adjusted *p* = 1.94E-03), and active transmembrane transporter activity (GO:0022804, FDR adjusted *p* = 2.40E-02). Additionally, reactome (REAC) pathway enrichment analysis demonstrated developmental processes including somitogenesis (FDR adjusted *p* = 7.43E-04), formation of paraxial mesoderm (FDR adjusted *p* = 1.48E-03), and gastrulation (FDR adjusted *p* = 8.78E-03). Immune-related pathways were also enriched in REAC analysis, including the ER–phagosome pathway (FDR adjusted *p* = 3.29E-03), antigen cross-presentation (FDR adjusted *p* = 5.22E-03), and MHC-mediated antigen processing and presentation (FDR adjusted *p* = 9.87E-03) (Supplementary Table 3).

## Discussion

In this study, *CASR* and *KLF2*/*KLF2-DT* were identified as being significantly associated with sPTB after FDR adjustment. The human *CASR* gene, located on chromosome 3q21.1, encodes the calcium-sensing receptor (CaSR), a G protein-coupled receptor (GPCR) that binds calcium ions to monitor and regulate blood calcium levels [21]. Beyond its systemic role in calcium homeostasis, *CASR* is also a key regulator of calcium signaling in organs such as the uterus and placenta [22]. *CASR* has been implicated in fetal development through its involvement in regulating fetal parathyroid hormone levels, bone, lung, and other organs or tissue development [23]. Additionally, both animal and human studies have identified *CASR* as playing a role in uterine contractions, cervical dilation, and key placental functions, including blood flow and hormone production [24]. *CASR* may regulate reproductive function by activating signaling pathways such as Mitogen-Activated Protein Kinase (MAPK) (e.g., ERK1/2, JNK) and Phosphoinositide 3-kinase /Protein Kinase B PI3K/AKT, both of which are critical for reproductive processes [25–28]. Through induction of eNOS and nitric oxide–mediated vasodilation, *KLF2*/*KLF2-DT* may play a critical role in maintaining uteroplacental perfusion, placental vascular integrity, and a low-inflammatory environment, all of which are essential for normal embryonic and fetal development [29, 30]. Although no studies have directly linked alterations in *CASR* and *KLF2*/*KLF2-DT* expression or signaling to the risk of sPTB, further research, including replication in an independent cohort and/or orthogonal experimental validation (e.g., targeted methylation assays), is needed to fully explore this potential association and to support strong biological conclusions.

In our study, we also observed increased methylation at CpG sites within *SNX14, HES7, PSMB8/PSMB9, TAP2, ATP2A3, SLC25A16, and GUCD1* and decreased methylation at CpG sites within *ALDH4A1 and SLCO3A1,* all of which are genes potentially involved in the pathophysiology of sPTB, although these differences were not statistically significant after adjustment for multiple comparisons, they are consistent with previous studies reporting that these genes are directly or indirectly associated with PTB. Although *SNX14* has not been found to be associated with PTB, one study reported that mutations or knockout of the *SNX14* gene are associated with childhood cerebellar atrophy and developmental delay and are linked to lysosome-autophagosome dysfunction [31]. In one study, *HES7* protein expression in the uteri and implantation sites of pregnant Balb/C mice was found to increase progressively with advancing gestational day [32]. Additionally, in a study conducted by Ware et al. involving amniotic fluid samples from 599 pregnant women in Connecticut, *PSMB8* was found to be upregulated [33]. This trypsin-like proteasome activity may originate from protein degradation in the chorio-decidua, and the observed increase was independent of gestational age but was associated with intra-amniotic infection and PTB [33]. *TAP2* was found to be highly expressed in human decidual stromal cells (DSCs) at the maternal–fetal interface, and its expression level was associated with fecundability [34]. Altered placental *SLCO3A1* gene expression is associated with intrahepatic cholestasis of pregnancy and may affect normal fetal development [35]. The other four CpG probes associated with sPTB in our study overlapped with genes such as *SLC25A16, ALDH4A1, ATP2A3, and GUCD1.* Although literature specifically linking these genes to pregnancy or sPTB is limited, these genes are known to be involved in mitochondrial energy production and cellular metabolism, calcium signaling and ion homeostasis, and biological pathways that have been implicated in placental function and fetal development, warranting further investigation. [36–39]

Given that male and female fetuses respond differently to adverse intrauterine conditions, we identified distinct maternal blood DNA methylation signatures associated with term and preterm births that exhibited sex-specific patterns, including hypomethylation of *KLF2* and *RPTOR* in women who delivered females and hypermethylation of cg00022064 in women who delivered males. These findings further support the presence of sexually dimorphic mechanisms in fetal development, as previously reported by Santos et al. [40] Interestingly, sex specific epigenetic regulation of *KLF2* in female endothelial cells as reported by Qin et al., has been shown to mediate sex-related differences in developmental outcomes, suggesting a potential mechanistic link to our observations [41]. Furthermore, differential methylation of *RPTOR* between female term and preterm infants aligns with animal studies showing that *RPTOR*, a key component of *mTORC1*, regulates oocyte growth and follicular development. In murine models, loss of *RPTOR* in oocytes activates compensatory *PI3K* signaling to preserve fertility, suggesting that epigenetic modulation of *RPTOR* in humans may similarly influence sex-specific reproductive and developmental outcomes, potentially contributing to PTB risk [42]. Although cg18473733 in *KLF2* and cg23063399 in *RPTOR* exhibited strong methylation signals, they did not remain statistically significant after correction for multiple comparisons, cg00022064, located in a noncoding region of the X chromosome, remained significant in males term versus preterm births. Collectively, these loci are biologically interesting given their potential relevance in sex-specific patterns or key functional contexts, such as placental vascular dysfunction, inflammatory pathways, and placental insufficiency in sPTB [30, 43]. No prior literature has linked *NSMF* or *PRPF38A* to pregnancy or sPTB, may warrant further validation in independent cohorts or inclusion in pathway-level analyses.

The enriched pathways identified in our analysis align closely with biological mechanisms implicated in the pathophysiology of PTB. In particular, the enrichment of proteasome-related functions suggests a role for proteasome-mediated antigen processing and protein turnover in the initiation of PTB. Supporting this association, Luo et al. demonstrated in a human placental study that altered expression of ubiquitin–proteasome system-associated genes is linked to sPTB and preterm premature rupture of membranes [44]. Additionally, Ware et al. reported that proteasome activity is present in amniotic fluid and is increased in inflammation associated PTB, further implicating proteasome dysregulation in inflammatory pathways contributing to PTB. [45]

## Conclusion

Our study identified potential epigenetic alterations associated with sPTB risk and highlighted candidate genes such as *CASR* and *KLF2*/*KLF2-DT*, as well as biological process include cellular transport and vascular regulation, including vasodilation. We also identified molecular functions involve enzymatic protein degradation (threonine peptidases) and energy-dependent transport of substances across membranes that may serve as potential predictors in pregnant Black women. Future research should examine larger samples of Black women and incorporate their risk factors of SDOH, including individual and structural racism, to better understand racial disparities in DNA methylation patterns. Larger, interdisciplinary studies are needed that explore the interactions between SDOH and epigenomic mechanisms underlying adverse birth outcomes.

## Limitations

Although our study includes a relatively large overall sample size compared to prior epigenetic research, the number of preterm cases remains limited, which restricts the generalizability of our findings. Additionally, the use of a single time-point blood sample limits the ability to evaluate dynamic, time-dependent changes in epigenetic modifications. The Illumina Infinium MethylationEPIC v2.0 assay contains only a subset of the CpG sites across the human genome. EWAS can be influenced by test statistic inflation; in our analysis, we observed moderate inflation, with a genomic inflation factor (λ) of 1.16. To assess the robustness of our findings, we performed a sensitivity analysis using the BACON method, which corrects for bias and inflation in test statistics. The results remained largely consistent after correction, supporting the robustness of our findings (Supplementary Fig. 1 and 2). Our study focused exclusively on maternal epigenetic signatures, which may not fully capture the infant’s epigenetic responses to maternal SDOH. Our sample included only Black women to examine differences among those at highest risk for PTB. We did not compare DNAm signatures across different racial groups as disparities have already been well documented between Black and White pregnant women [46, 47]. Future research should incorporate longitudinal biospecimen collection and larger, more diverse cohorts of Black women and their infants, to more comprehensively examine the impact of SDOH, including both individual- and structural-level racism, on DNA methylation patterns in the mother-infant dyads. These future studies will also for a more comprehensive understanding of how social exposures such as racism and discrimination result in higher rates for many Black women, but not all.

## Supplementary Information

Additional file 1.

Additional file 2.

## Data Availability

The data generated and/or analyzed during the current study are available from the corresponding author on reasonable request. Other deidentified nuMoM2b study data is stored in the NICHD Data and Specimen Hub (DASH) website: https://dash.nichd.nih.gov/. The EWAS summary statistics will be available in the following link upon publication: 10.5281/zenodo.19668610.
