# Supplementary figures and images for: Epigenome-wide DNA methylation and spontaneous preterm birth among pregnant black women
Source: Clin Epigenetics. 2026 May 24;18:146. doi: 10.1186/s13148-026-02151-8 (PMC13377820; doi:10.1186/s13148-026-02151-8)

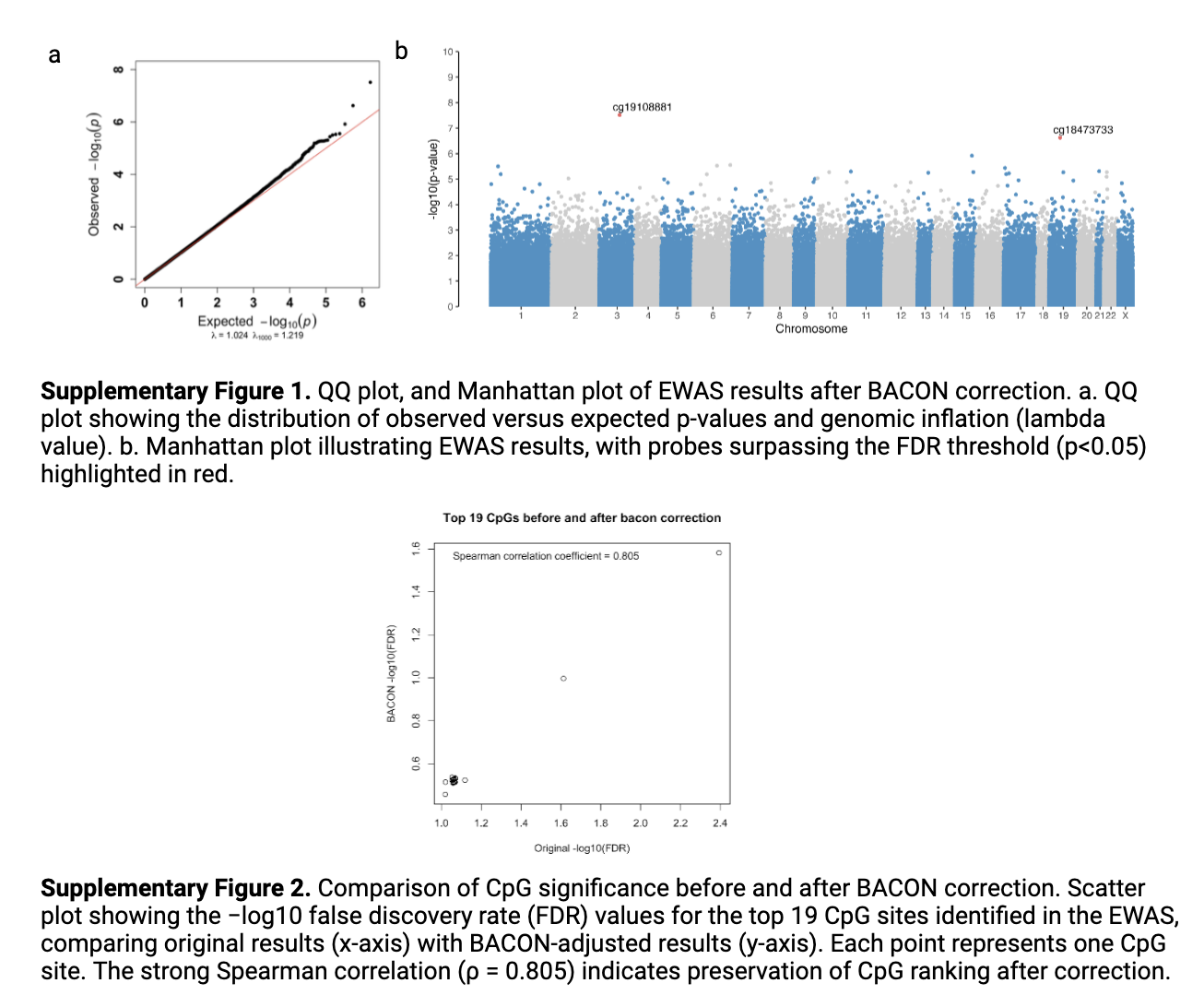

Supplement: Supplementary file 1 — Additional file 1. [file 13148_2026_2151_MOESM1_ESM.docx]
