# Supplementary material for: Epigenome-wide DNA methylation and spontaneous preterm birth among pregnant black women
Source: Clin Epigenetics. 2026 May 24;18:146. doi: 10.1186/s13148-026-02151-8 (PMC13377820; doi:10.1186/s13148-026-02151-8)
Supplement: Supplementary file 2 — Additional file 2. [file 13148_2026_2151_MOESM2_ESM.docx]

**Supplementary Table 1. Differentially methylated probes among pregnant Black women who delivered female preterm compared with female term infants.**

*Note.* Δβ, probe delta beta. Chromosome positions are shown relative to the human reference genome (hg38).

| Probe ID | Chromosome | Position | Gene | Annotatr Annotation | Gene region | Δβ | p value | Adjusted p value |
| --- | --- | --- | --- | --- | --- | --- | --- | --- |
| cg18473733 | chr19 | 16326551 | - | KLF2 | - | -0.016 | 1.34E-07 | 0.089 |
| cg23063399 | chr17 | 80613890 | - | RPTOR | - | -0.023 | 4.05E-07 | 0.089 |
| cg06077979 | chr9 | 137454543 | - | NSMF | - | 0.019 | 4.18E-07 | 0.089 |
| cg19776520 | chr1 | 52404175 | PRPF38A | PRPF38A | TSS1500 | 0.005 | 4.22E-07 | 0.089 |

**Supplementary Table 2. Differentially methylated probes among pregnant Black women who delivered male preterm compared with male term infants.**

*Note.* Δβ, probe delta beta. Chromosome positions are shown relative to the human reference genome (hg38).

| Probe ID | Chromosome | Position | Gene | Annotatr Annotation | Gene region | Δβ | p value | Adjusted p value |
| --- | --- | --- | --- | --- | --- | --- | --- | --- |
| cg00022064 | chrX | 41046880 | - | - | Intergenic | 0.045 | 5.09E-10 | <0.001 |

**Supplementary Table 3. Functionally enriched gene ontology (GO) and pathways.**

| **Term ID** | **Source** | **Term Name** | **Intersection of Genes** | **FDR adjusted P value** |
| --- | --- | --- | --- | --- |
| GO:1901653 | GO:BP | cellular response to peptide | CASR, KLF2 | 4.91E-04 |
| GO:0046967 | GO:BP | cytosol to endoplasmic reticulum transport | ATP2A3, TAP2 | 1.89E-03 |
| GO:0042311 | GO:BP | vasodilation | CASR, KLF2 | 6.88E-03 |
| GO:0099170 | GO:BP | postsynaptic modulation of chemical synaptic transmission | SNX14, FBXO2 | 9.48E-03 |
| GO:0003018 | GO:BP | vascular process in circulatory system | CASR, KLF2, ATP2A3, SLCO3A1 | 1.27E-02 |
| GO:1990111 | GO:CC | spermatoproteasome complex | PSMB9, PSMB8 | 3.78E-04 |
| GO:0019774 | GO:CC | proteasome core complex, beta-subunit complex | PSMB9, PSMB8 | 2.07E-03 |
| GO:0036477 | GO:CC | somatodendritic compartment | CASR, SNX14, FBXO2, RTN4RL1 | 3.40E-03 |
| GO:0005839 | GO:CC | proteasome core complex | PSMB9, PSMB8 | 9.50E-03 |
| GO:0070003 | GO:MF | threonine-type peptidase activity | PSMB9, PSMB8, GGT1 | 5.08E-05 |
| GO:0004298 | GO:MF | threonine-type endopeptidase activity | PSMB9, PSMB8 | 1.94E-03 |
| GO:0022804 | GO:MF | active transmembrane transporter activity | SLC25A16, ATP2A3, TAP2, SLCO3A1 | 2.40E-02 |
| REAC:R-HSA-9824272 | REAC | Somitogenesis | HES7, PSMB9, PSMB8 | 7.43E-04 |
| REAC:R-HSA-9793380 | REAC | Formation of paraxial mesoderm | HES7, PSMB9, PSMB8 | 1.48E-03 |
| REAC:R-HSA-1236974 | REAC | ER-Phagosome pathway | PSMB9, TAP2, PSMB8 | 3.29E-03 |
| REAC:R-HSA-1236975 | REAC | Antigen processing-Cross presentation | PSMB9, TAP2, PSMB8 | 5.22E-03 |
| REAC:R-HSA-9758941 | REAC | Gastrulation | HES7, PSMB9, PSMB8 | 8.78E-03 |
| REAC:R-HSA-983169 | REAC | Class I MHC mediated antigen processing & presentation | FBXO2, PSMB9, TAP2, PSMB8 | 9.87E-03 |

**Supplementary Table 4. Differentially methylated probes among pregnant Black women who delivered preterm infants compared with term infants before and after BACON correction.**

| Probe ID | Chromosome | Position | Gene | Gene Region | Δβ | p value | Adjusted  p value | BACON Correction | | |
| --- | --- | --- | --- | --- | --- | --- | --- | --- | --- | --- |
|  |  |  |  |  |  |  |  | test statistic | P value | Adjusted p-value |
| cg19108881 | chr3 | 122183663 | CASR | TSS1500 | 0.012 | 4.93E-09 | 0.004 | 5.537 | 3.07E-08 | 0.026 |
| cg18473733 | chr19 | 16326551 | KLF2, KLF2-DT | – | -0.012 | 5.87E-08 | 0.025 | -5.167 | 2.38E-07 | 0.099 |
| cg24569447 | chr15 | 88769564 | – | – | 0.005 | 2.72E-07 | 0.076 | 4.854 | 1.21E-06 | 0.300 |
| cg25689730 | chr6 | 85593744 | SNX14, SYNCRIP | exon_1 | 0.002 | 7.35E-07 | 0.087 | 4.672 | 2.99E-06 | 0.300 |
| cg04872402 | chr1 | 11645926 | FBXO2 | – | 0.003 | 7.85E-07 | 0.087 | 4.659 | 3.17E-06 | 0.300 |
| cg12798411 | chr6 | 167235896 | HPAT5, LOC102725048 | – | -0.013 | 8.61E-07 | 0.087 | -4.684 | 2.81E-06 | 0.300 |
| cg03098704 | chr17 | 1957116 | RTN4RL1, LOC105371486 | – | -0.008 | 1.15E-06 | 0.087 | -4.630 | 3.66E-06 | 0.300 |
| cg25826287 | chr21 | 36073515 | SETD4 | – | 0.016 | 1.28E-06 | 0.087 | 4.568 | 4.93E-06 | 0.300 |
| cg15165108 | chr10 | 68527740 | SLC25A16 | TSS1500 | 0.008 | 1.40E-06 | 0.087 | 4.551 | 5.35E-06 | 0.300 |
| cg12328625 | chr13 | 99865146 | CLYBL, CLYBL-AS3 | – | 0.007 | 1.48E-06 | 0.087 | 4.540 | 5.63E-06 | 0.300 |
| cg09601704 | chr17 | 8124109 | HES7 | TSS200 | 0.002 | 1.56E-06 | 0.087 | 4.529 | 5.93E-06 | 0.300 |
| cg05919744 | chr11 | 2902928 | SLC67A1, SLC67A1-AS | – | -0.027 | 1.64E-06 | 0.087 | -4.562 | 5.07E-06 | 0.300 |
| cg12120292 | chr17 | 3963933 | ATP2A3 | – | 0.002 | 1.70E-06 | 0.087 | 4.513 | 6.40E-06 | 0.300 |
| cg24031377 | chr6 | 32842249 | PSMB8, PSMB8-AS1, PSMB9, TAP2 | exon_4 | 0.008 | 1.72E-06 | 0.087 | 4.510 | 6.47E-06 | 0.300 |
| cg05111081 | chr15 | 91882266 | SLCO3A1 | – | -0.006 | 1.73E-06 | 0.087 | -4.552 | 5.31E-06 | 0.300 |
| cg26144263 | chr22 | 24968597 | KIAA1671 | – | -0.009 | 1.74E-06 | 0.087 | -4.551 | 5.34E-06 | 0.300 |
| cg00381057 | chr19 | 29873781 | – | – | -0.005 | 1.77E-06 | 0.087 | -4.547 | 5.43E-06 | 0.300 |
| cg12092708 | chr1 | 18902651 | ALDH4A1 | TSS200 | -0.004 | 2.13E-06 | 0.096 | -4.512 | 6.43E-06 | 0.300 |
| cg11895717 | chr22 | 24555095 | GUCD1, SNRPD3 | exon_1 | 0.001 | 2.19E-06 | 0.096 | 4.463 | 8.07E-06 | 0.354 |

*Note*. Chromosome positions are shown relative to the human reference genome (hg38). The *Δβ* values refer to the difference between DNA methylation (*β*-values) in cases compared to controls (e.g., 0.012 indicates that pregnant Black women who delivered preterm infants show a 1.2% increase in DNA methylation compared to term infants). Adjusted p value refer to FDR adjusted *p* values.
